# Coordinating Mental and Physical Health Care in Rural Australia: An Integrated Model for Primary Care Settings

**DOI:** 10.5334/ijic.3943

**Published:** 2018-06-05

**Authors:** Scott J. Fitzpatrick, David Perkins, Tonelle Handley, Dale Brown, Teresa Luland, Eamonn Corvan

**Affiliations:** 1 Centre for Rural and Remote Mental Health, The University of Newcastle, Orange East, NSW 2800, AU; 2 Mudgee Community Health Centre, Mudgee, NSW 2850, AU

**Keywords:** mental health, primary care, integration, rural health, Australia

## Abstract

**Introduction:**

The ‘GP Clinic’ provides primary health care to people using community mental health services in a small town in Australia. This article examines the factors that have driven successful integration in this rural location.

**Methods:**

A multiple methods case study approach was used comprising service record data for a 24 month period and semi-structured interviews with sixteen staff members associated with the integrated rural service model.

**Results:**

Processes and structures for establishing integrated care evolved locally from nurturing supportive professional and organisational relationships. A booking system that maximised attendance and minimised the work of the general practice ensured that issues to do with remuneration and the capacity for the general practitioner to provide care to those with complex needs were addressed. Strong collaborative relationships led to the upskilling of local staff in physical and mental health conditions and treatments, and ensured significant barriers for people with mental illness accessing primary care in rural Australia were overcome.

**Conclusions:**

Integrated physical and mental health service models that focus on building local service provider relationships and are responsive to community needs and outcomes may be more beneficial in rural settings than top down approaches that focus on policies, formal structures, and governance.

## Introduction

There is strong evidence that the integration of primary health and specialist mental health care is positively associated with improved quality and continuity of care, reduced health inequalities, and that it may reduce health care costs [1,2]. Integrated care can take different forms based on different conceptual frameworks. What is essential is its commitment to overcoming fragmented care, and to meeting complex care needs through ongoing and co-productive partnerships [3].

In Australia, mental health policies focus on primary care interventions because of the widespread accessibility of general practitioners, their potential to identify and improve treatment rates, and their ability to treat co-morbid mental and physical health problems [1,4]. This is particularly important in rural communities where there is a shortage of specialist mental health services [5].

However, concerns have been raised about general practitioners’ low self-efficacy in diagnosing and treating patients with both high and low prevalence mental health conditions, as well as their ability to address physical health problems in people with mental disorders [4]. Those with severe and persistent mental illness such as schizophrenia also experience difficulties in accessing primary care services. This may be due to the primary care setting being ill-equipped to deal with the needs of this population [6]. For example, booking systems and waiting areas can be hard to navigate and services may not be available in a timely manner [7,8]. In addition, high non-attendance rates together with the time required to address the complex set of medical concerns experienced by people with severe and persistent mental illness mean that holistic person-centred physical and mental health care may not be financially viable in general practice under current funding arrangements [6].

Governance structures, workforce shortages, and professional roles and culture can also constrain clinical and service level integration [9]. Limited access to psychiatrists continues to impair the care of patients with severe and persistent mental illness, and specialist input is necessary to enhance general practitioners’ skills and confidence to manage complex mental health problems [10]. Moreover, integration at the organisational level does not always lead to improvements in the way health care professionals work together [11]. Despite a willingness from general practitioners and community mental health teams to collaborate, research has highlighted a number of tensions with regards to roles and capabilities, referral processes, and levels of support [12].

To maximise service effectiveness for people with severe and persistent mental illness, approaches that concentrate on integration around the patient pathway and frontline team are thought to have several advantages. They are designed within local resource capacity limits, and so are more likely to maximise practitioner knowledge and involvement and be financially and clinically sustainable [13,14].

In Australia, research on integrated service models has focused on the evaluation of short term pilot projects, paying insufficient attention to local contexts in which problems and services are located. There is little research that has examined successful integrated service models for people with severe and persistent mental illness. However in rural Australia, where policy, health systems, and alignment of incentives favour normal ‘fragmented’ services, the analysis of well-established service models is informative and can be used to inform innovative integrated service design and delivery [15]. This paper is novel because it examines integrated care in an established mainstream rural service developed without additional pilot funding or changes in systems or incentives.

## Methods

The analytic objective of the research was to describe and understand the components, practices, and processes that have driven successful integration of primary and community based specialist care (the ‘GP Clinic’) for people with severe and persistent mental illness in a small rural town in New South Wales, Australia. A multiple methods case study approach was employed to address questions about service activity, as well as to describe the objectives of the GP Clinic, its management, benefits, and the keys to its continued operation.

### Research setting and participants

In Australia, hospital services are the responsibility of the states and medical services, whether they are provided by general practitioners or specialists, are provided in the private sector and subject to a schedule of reimbursements to patients through a voluminous schedule of payments called the Medicare Benefits Schedule. Doctors are free to charge a co-payment on top of this reimbursement and where the market permits they often do. The Medicare System provides universal access to free or subsidised health care with one proviso, general practice and medical services are poorly distributed so that access is good in capital cities and major centres but it becomes harder to see a doctor or allied health provider in rural and remote areas. There is also a large private insurance system which focusses on elective care, and the federal government charges penalties to those aged over 30 who do not take private health cover.

Broadly speaking, 30% of Australians live in regional, rural, and remote areas, with the majority of these living close to the coast. This leaves a large interior with small communities and low population densities. The community described in this paper is one such town of approximately 11,000 people with mining, viticulture, and tourism industries. It is a pleasant and popular town but the small population means that there is a shortage of specialists in most fields. Mental health services are provided by general practitioners who tend to provide pharmaceutical treatments, a state funded community mental health specialist service, and some private psychological services which are supported by time-limited subsidies to patients. Two visiting psychiatrists visit the community mental health service one day each on alternate weeks or four days per month. There are also some community organisations who provide mental health and related welfare and other services. The nearest mental health inpatient unit is two hours away by road.

### Description of the care practice

The GP Clinic was established in 2007 in response to concerns about poor access to primary health care for patients of the community mental health service. It provides comprehensive primary health care to patients through a regular monthly clinic run by a local general practitioner with appointments managed by the community mental health team. The community mental health team – a multidisciplinary team consisting of mental health nurses, a social worker and psychologist – is responsible for organising patient appointments and assisting patients to attend (including providing transport if needed). With the consent of the patient, a community mental health team member accompanies patients during their consultation with the general practitioner and during clinical evaluations and reviews with the visiting psychiatrist. Patients are also able to bring family members and/or informal carers to consultations with the general practitioner.

Upon acceptance by the community mental health service, new patients without a general practitioner are booked into the GP Clinic for full blood tests, liver function tests, and other routine testing. They are also booked in to see the visiting psychiatrist who provides a diagnostic framework and management plan to inform the patient’s Mental Health Treatment Plan. In consultation with the psychiatrist, community mental health team, patient, and family/carer(s), the general practitioner then develops a care plan that lists the patient’s main issues/needs; the corresponding goals, treatments, and referral pathways for each; and arrangements for crisis intervention and/or relapse prevention. Regular case reviews based on acuity and culminating in a major clinical review every three months are conducted with input from the GP Clinic, community mental health team, and drug and alcohol services. This ensures joint care planning and continuity of care for patients with comorbid physical, mental health, and alcohol and/or other drug problems.

### Data collection

This study used a multiple methods approach. To provide an overview of the activity of the GP Clinic, clinical data was collected from service records for each patient who attended the GP Clinic over a 24 month period to September 2015. Data items included: patient characteristics (date of birth, gender, time as a patient of the community mental health team, primary mental health diagnosis), GP Clinic attendances (date of referral to clinic, number of clinics attended in two year period, reason for attending the clinic), and management of patient (client referrals, arrangements for ongoing access to primary care).

We also invited health care providers and other staff associated with the GP Clinic: community mental health team members (n = 11), general practitioners (n = 2), visiting and consultant psychiatrists (n = 2), and practice managers (n = 1) to participate in face-to-face, semi-structured interviews. These informants were purposively selected based on their involvement in the development, management, and/or their participation in the GP Clinic. Sixteen participants were interviewed between December 2015 and April 2016. The study was approved by the Greater Western Area Health Service and University of Newcastle Human Research Ethics Committees.

### Data analysis

Interviews were recorded on a digital voice recorder, transcribed verbatim, and de-identified. A Framework Method was used to ensure systematic and rigorous analysis of the data. The Framework Method is a flexible analytic tool used to classify and organise data according to key themes and concepts [16,17]. This approach ensured interpretation remained grounded in the data while enabling synthesis of recurring patterns of responses to build on existing theoretical understandings of integrated care.

For the quantitative analysis, participants were categorized by their primary diagnosis, and characteristics of their GP Clinic attendance patterns were explored. Attendance patterns were also explored by demographic characteristics, with t-tests being used for categorical variables and correlations used for continuous variables.

## Results

### Service data

Over the two-year period, 65 individuals attended the GP Clinic. There were similar numbers of males and females, with the majority of attendees aged under 55 years (see Table 1). The most common diagnosis was a psychotic disorder (35% of patients), followed by psychiatric comorbidity (20%) or a mood disorder (17%). Approximately one-third of attendees have been community mental health patients for more than five years.

**Table 1:** Attendance patterns of the GP Clinic over previous 2 year period.

Characteristic	Mental health diagnosis					

	Psychotic disorder	Mood disorder	Substanceuse disorder	Psychiatric comorbidity	Other	Total
	n = 23	n = 11	n = 8	n = 13	n = 10	n = 65

**Age (years)**						

18–34	7	5	5	1	6	24
35–54	13	5	2	9	2	31
55+	3	1	1	3	2	10
**Gender**						

Male	10	7	7	7	4	35
Female	13	4	1	6	6	30
**Client of Community Mental Health Service (years)**						

0–4	11	7	7	9	7	41
5+	12	4	1	4	3	24
**GP Clinic attendance**						

1–2 times	9	6	4	5	6	30
3–9 times	9	3	4	6	3	25
10+ times	5	2	0	2	1	10
Did not attend	1	1	2	1	4	9
**Ongoing access to GP services**						

GP Clinic	14	1	1	5	2	23
GP Independently	5	6	3	4	7	25
Moved	3	2	1	4	1	11
Other	1	2	3	0	0	6

**Figure 1:**
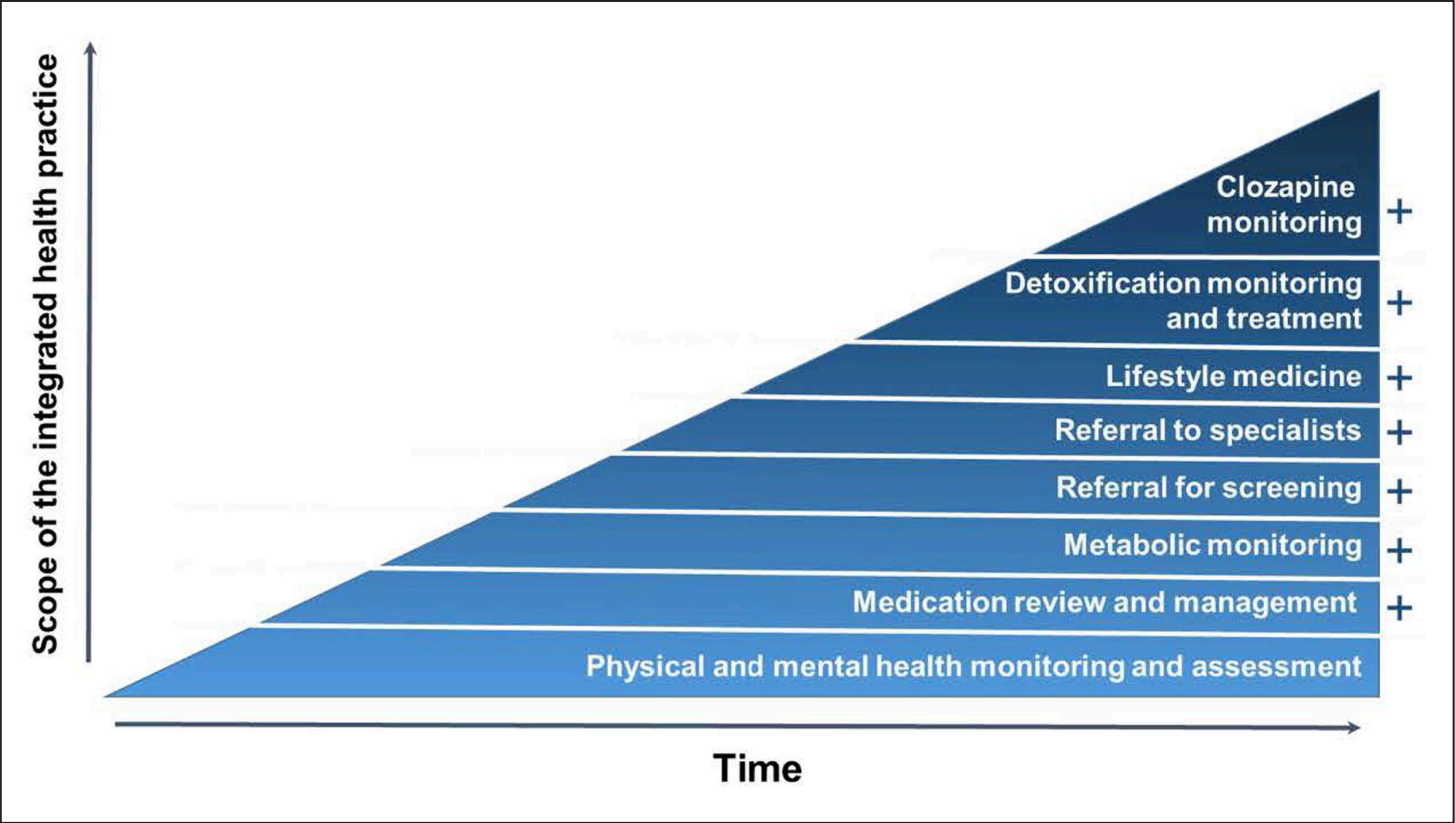
Increasing scope of GP Clinic over time.

On average, patients attended 5.1 sessions (±5.7) over the two-year period (range 1–23). Females typically attended more sessions (7.0 ± 6.8) than males (3.5 ± 4.0; t(63) = –2.62, *p* = .011). Older patients also attended more sessions, although the effect size was small (r = 0.29, *p* = .018). The greatest number of sessions was attended by those with a psychotic disorder (6.2 ± 5.8), and the fewest by those with a substance use disorder (2.1 ± 1.2), although there were no significant differences in the number of sessions attended between diagnoses. Of the 341 appointments that were booked in total over the two-year period, only nine of these resulted in a non-attendance. The services provided to patients during the study period are outlined in Figure 1. These reflect the increasing scope of the GP Clinic over time.

### Provider views

This paper reports on three themes regarding the integration of primary health and specialty mental health care within primary care settings. The first theme, *local and broader health structures*, outlines the micro and macro level system structures required to support the integrated care model. The second theme, *nurturing interpersonal relationships*, highlights the importance of relational aspects for supporting change and fostering collaborative and patient-centred practice. Finally, *benefits of collaborative and integrated practice* encapsulates diverse perspectives on the value to health providers and patients of the model.

### Local and broader health structures: ‘it’s a simple model… just need a mental health team and a GP’ [30]

Several participants spoke of the simplicity of the integrated model, yet several factors were key to its operation and sustainability. Time and capacity for the general practitioner to provide care to those with complex needs and remuneration were described as important incentives.

“I don’t think there’s a lot of extra money out there for people to actually take on things like mental health.” [31].

“GPs are under supplied in the bush and they’re overworked, so they can be more picky about their patients… [and] can choose higher paying patients that are much more likely to attend” [60].

The community mental health team were aware of these challenges and a booking system was introduced that minimised work for the general practice and maximised attendance.

“They [general practice] blot out a whole day for the mental health team and usually one person on our team will organise those clients, and it’s usually prioritised to who needs it the most on the day” [46].

“Our non-attendance would be more if we didn’t have [community mental health team member] doing it” [54].

This system addresses both the concerns of the general practice with regard to non-attendance, but also patient concerns about access and the difficulty of navigating primary care in rural areas.

“We try to accommodate them with regards to times, to fitting around when they can make it in. If there’s transport issues or if there’s childcare issues we will try and work with them to try and fit them in in an appropriate time, and we give a double appointment because we actually know that a lot of mental health clients – they’re anxious, they’re slow to start and then when you get them going, they’re more comfortable and they find it more beneficial” [38].

Another distinctive feature of the GP Clinic is that attendance by a community mental health team member is normal practice.

“If we’ve got somebody booked in, their own case manager tries to sit in with them which helps to reinforce the continuity of service and, again, it gives the team ownership” [38].

Aside from the time required to manage and attend monthly appointments, the GP Clinic operated within existing community mental health team resources. However, the successful operation of the GP Clinic was dependent on current staffing levels. Outreach to smaller towns in the catchment area, large patient caseloads, and small teams where staff leave arrangements have considerable impacts means schedules need to be tightly coordinated.

“Not everyone is working full-time so there’s lots of chopping and changing and it does get difficult because of short staffing issues” [28].

“We don’t have as many resources from within the drug and alcohol services to participate in the Clinic and on a one-to-one basis along with the GP as we’d probably like” [38].

While current staffing levels did not directly impact sustainability of the GP Clinic, it did hamper operations with regards to timely patient record keeping and sharing – a problem exacerbated by the use of different records management systems by mental health and drug and alcohol workers – as well as future planning. The precariousness of Medicare funding was also a potential barrier to its ongoing financial viability for the general practitioner.

“It’s most probably still very viable. But if Medicare doesn’t increase the rebate in the next couple of years I can see that it could be that [general practitioner] would be asked to do it for less than what he would be doing if he saw private patients for the day” [54].

In practice, however, the basis of the GP Clinic model was viewed as simple and something that could be replicated wherever relationships between community mental health and general practitioners were good.

“[For] anybody trying to do this, don’t put it in as a full day’s clinic or something, just take it – one worker who’s got a good relationship with a GP, book in a couple of their clients together, do joint consults and that effectively is the – that’s the cell of a GP Clinic. It starts simple and then it actually can grow” [38].

### Nurturing interpersonal relationship: ‘health care provision is about human relationships’ [55]

There was a perception among participants that strong interpersonal relationships were pivotal to the realisation of successful collaborative practice, and that any attempt to replicate the service model required the building of a trusting and supportive environment between the community mental health team, local general practitioners, and visiting psychiatrists.

“We have nurtured a relationship with all the GPs, and the GP Clinic became part of the extension of that relationship…” [55].

Processes and structures for establishing local joint working arrangements, therefore, evolved locally out of these relations rather than out of formal organisational structures or policies.

“What works with the GP Clinic [is] it’s in the general practice and substantially by design of the GP and we fit in around it. It’s grown organically from that” [60].

From the community mental health team’s perspective, recognition of the role and expertise of the general practitioner was important to creating a culture conducive to integrated care.

“What I try to do in all of my interactions with the GP is to make him, I hope, feel that I value his contribution towards the welfare of any of the patients that we’re jointly looking after…. How you handle the interpersonal relationship is terribly important because everyone wants to be made to feel that they have an important role” [55].

This fostering of close working relations between the community mental health team and general practice had the effect of allowing community mental health patients to develop longer-term relationships with the general practitioner, thus overcoming previous barriers to accessibility experienced by those with severe and persistent mental illness such as low self-confidence, long waiting lists, and difficulties with booking systems and finding empathic physicians.

“That’s three clients now that started with a GP Clinic and, then, as they became well, have dropped out of the GP Clinic but continued to see that GP. And I think for those three particularly, having that support really helped them” [34].

“We make the initial contact for them, but after a period of time they then maybe decide I don’t need the team to do it for me, I can do it myself so they may make individual appointments” [53].

The presence of active, visible, and longstanding leaders has undoubtedly played an important role in relationship building and, in turn, the success and sustainability of the GP Clinic. Apart from a change in general practitioner operating the GP Clinic, staffing has remained consistent since its establishment in 2007. Participants attributed this to a strong attachment to the town and felt that this contributed to the stability of the GP Clinic and a shared sense of purpose.

“Once people come to [town] they love it and don’t want to leave… Compared to other places that I’ve worked people are really dedicated to their work, they want to be here and they want to do well at what they do, and I suppose there’s some people like that in other services, but not everyone… works together to the same goal” [46].

### Benefits of collaborative and integrated practice: ‘there’s satisfaction to be gained by helping people who are falling through the cracks’ [31]

The development of professional and organisational relationships led to clear collaborative advantages, and the mutual benefits of collaboration were seen by many as reasons for the GP Clinic’s ongoing operation. For the community mental health team, these benefits included the physical health care of patients.

“Our psychiatrists find it a benefit because they’re seeing their recommendations for physical follow up, physical health conditions being followed up and, therefore, their relationship with the GPs are working a lot better as well” [46].

Care planning and treatment were also greatly improved by enabling direct and more effective communication between the general practitioner, community mental health team, and patients.

“A lot of GPs [and community mental health staff] work part-time so you’re often playing tag… trying to contact and liaise with GPs, whereas with a GP Clinic you’ve got everyone in the room on the same page. I think that’s a great way to care plan” [34].

“I think clients appreciate both being able to see their doctor and the mental health worker at the same time, because potentially that’s one less appointment and everyone knows what’s going on” [30].

It also had organisational and cost-saving benefits by ensuring more efficient practices for managing patients with severe and persistent mental illness and those with mild to moderate mental health problems.

“This model is particularly powerful in the community because it allows our patients better access to a GP with obvious positive effects on their physical health, as well as being easy to discharge patients off the books so we can see more acute and disturbed patients” [60].

A further benefit of collaborative practice was the upskilling of local clinical staff. The community mental health team were more aware of physical health conditions and treatments, while the general practitioner’s knowledge of managing patients presenting with severe and persistent mental illness was improved.

“This is not stuff that’s mainstream mental health but at the same time it helps upskill staff with regards to some of these issues. And if they’re seeing another client they’re actually able to say, ‘well, why don’t you go to the doctor because they’ve actually got some stuff they can do for that’” [38].

“I would hope that [my] skills in terms of dealing with mental health… have been, hopefully, a smidgen improved as a result of partaking in the Clinic” [30].

Building on this confidence and increased trust between general practitioner and visiting psychiatrists, professional roles were revisited to enable the general practitioner to address service shortcomings in the local community around drug and alcohol detoxification and clozapine treatment and monitoring.

“[We] had some people that we wanted to start on clozapine, but were just so fearful about going into a psychiatric hospital to start. And I think [visiting psychiatrist] rang [general practitioner] and said, “Look, if I supported you during that period of time, with the knowledge and all that sort of stuff, would you be prepared to do it?”” [50].

“Having that access to the GP Clinic and people wanting to detox at home… I think that provides safer monitoring” [34].

In addition to meeting patient and community needs, both general practitioners and the community mental health team spoke of the impacts of the service model on their quality of working life.

“The mental health clients, I guess, from a medical point of view, have a notable amount of pathology in terms of people with real illnesses like bipolar and schizophrenia. And such things, I think, are both a challenge and quite rewarding. I think that medical people like helping people who have real medical conditions. It’s something that’s interesting, and a rewarding reason for doing the Clinic” [30].

“You’re actually achieving something with your work. So you’re seeing clients improve and get better rather than declining, and that makes you want to come to work and do your job and keep going” [46].

## Discussion

Government programs such as the Better Access to Mental Health Care initiative have given general practitioners greater scope in the management of mental health patients in Australia, primarily by referring to psychologists and other allied health professionals [18]. Recent debates over the appropriateness of general practitioners managing severe and persistent mental illness without input from psychiatrists, however, suggests that improving specialist support through activities such as shared case management may be just as important as improving referral mechanisms [10]. The present study has provided insights into the development of a rural health service model in a small town that provides primary and specialist mental health care to patients with severe and persistent mental illness. While this study did not examine the views of service users, the benefits of this model for practitioners included improved professional support, increased knowledge of both physical and mental health conditions, the provision of high quality patient–centred care with potentially better patient outcomes, and more efficient use of available resources.

The wide-ranging and multiple set of ideas and principles that contribute to integrated care and the different forms that it can take make prescriptive models inappropriate [19]. Data from this study confirm the importance of addressing the organisational and professional challenges that have traditionally hampered cross-professional and cross-sectoral collaboration such as inconsistent service protocols, funding arrangements, and organisational and professional roles and cultures [12,20]. For example, new leadership roles in the community mental health team to manage monthly bookings ensured that the service was financially viable for the participating general practitioner by maximising attendances. While relinquishing control of the booking system presented an initial challenge for the general practice, the benefits were evident in the low non-attendance rates of the GP Clinic.

The redefining of professional roles and responsibilities and the fostering of a shared culture were important to the development and formalisation of collaborative working arrangements [21]. The participation of community mental health staff in separate patient consultations with both visiting psychiatrists and the general practitioner marked a radical departure from traditional hierarchies and service models that revolve around the needs of health practitioners, to one that placed the patient firmly at the centre. This led to more effective and efficient case management and referral pathways, the building of stronger therapeutic relations, and improved continuity of care for patients.

Once established, the relative simplicity of processes and structures required to run the GP Clinic has allowed partners to concentrate on the purpose and priorities of the service. A shared vision of service outcomes and the generation of mutually recognised benefits are acknowledged as important indicators of whether a chosen approach is working [20,22]. Starting with the simple goal of improving access to primary care for those with severe and persistent mental illness, the GP Clinic has built on its success to address more complex issues such as drug and alcohol treatment and clozapine prescribing. In doing so, it has provided additional benefits to patients, health professionals, the health service, and the community.

The quantitative analysis highlighted several interesting findings, particularly related to patients living with psychotic disorders. Patients with this diagnosis were the most likely to have been a client of the community mental health team for five or more years. They attended the highest number of sessions during the period of data collection, although this effect was not statistically significant and further research in a larger sample is necessary to confirm this finding. In addition, there was only one instance of non-attendance to an appointment during this time. People living with psychotic disorders are often among the most difficult to engage in primary care services due to the nature of their symptoms [6,23]. However, our findings suggest a level of acceptability of the GP Clinic for patients with this diagnosis. In addition, it is often difficult for individuals living with psychotic disorders to receive appropriate physical health care due to inequalities in medical care in rural settings [15]. Hence, an additional benefit of the GP Clinic is that it provides appropriate services to this vulnerable group who otherwise may be unable to access primary care.

The wider implication of this study for rural integrated mental and physical health care models in Australia is the degree to which locally-driven co-designed service models that are responsive to local needs, resources, and that build on existing clinical relationships are more effective drivers of sustainable practice transformation towards multidisciplinary care than ‘top down’ approaches that are constructed by government. Programs such as consultation-liaison psychiatry to general practitioners and the Mental Health Nurse Incentive Program are examples of two recent programs aimed at improving general practitioner capacity to deliver high quality mental health care – the former through the provision of psychiatric consultations to general practitioners and the latter through funding general practices to engage mental health nurses. Both programs have been positively received with general practitioners reporting improved support and confidence in managing patients with mental health problems [24,25]. However, the instrumentalisation of professional roles within program guidelines that restricts psychiatric and mental health nursing practice to limited roles of diagnosis and management may curtail the capacity of these programs to deliver integrated physical and mental health care in a multidisciplinary and holistic framework [26]. In the case of the Mental Health Nurse Incentive Program, co-location in the same clinic did not always lead to the forging of consultative and team-based roles but often reinforced existing power relations between disciplines [26]. It is also unclear to what extent these programs facilitated general practitioner involvement in the integrated care of patients with severe and persistent mental illness being treated by community mental health services.

## Conclusion

This integrated service model is unusual in the Australian health system since it is based on local co-design rather than state or national pilot programs, has been established for more than ten years, and operates within normal financial arrangements. Unfortunately, it has proven difficult to build similar services in comparable neighbouring communities. While strong leadership, staff stability, and place-based factors such as the amenity of the rural locale play an important role, findings from this study show the importance of clear outcomes and interpersonal relationships in the building of locally relevant and sustainable integrated care services for people with severe and persistent mental illness. It also highlights the benefits of co-design versus standard solutions in which collaborative engagement generates value for community health services, professionals, and patients. From this perspective, it is the “process of emerging partnership synergy” and not clearly defined program structures that are the key drivers of the GP Clinic’s success [27]. This may explain the challenges facing health administrators and policy makers in promoting the model more widely, and indicates a need to support and fund adaptive local co-designed solutions over conventional approaches to program design.
